# Clinical efficacy of *Azadirachta indica* based herbal mouthwash in treating the hypersensitivity of teeth

**DOI:** 10.12669/pjms.40.10.9826

**Published:** 2024-11

**Authors:** Afsheen Mansoor, Emaan Mansoor, Nehal Amir, Khadim Hussain

**Affiliations:** 1 Afsheen Mansoor, PhD (Medical Microbiology, Dental Materials, Nanotechnology), Associate Professor, Quaid-i-Azam University, Islamabad, Pakistan. Department of Dental Material Sciences, School of Dentistry, Shaheed Zulfiqar Ali Bhutto Medical University, Islamabad, Pakistan; 2 Emaan Mansoor, Islamic International Dental College, Riphah International University, Islamabad, Pakistan; 3 Nehal Amir, Post Graduate Resident, Department of Dental Material Sciences, School of Dentistry, Shaheed Zulfiqar Ali Bhutto Medical University, Islamabad, Pakistan; 4 Khadim Hussain, Statistical Analysist, Department of Statistics, Islamia University, Bahawalpur, Pakistan

**Keywords:** *Azadirachta indica*, Herbal, Mouthwash, Tooth sensitivity

## Abstract

**Objective:**

To evaluate the effectiveness of Azadirachta indica based Herbal mouthwash to treat tooth sensitivity in patients.

**Method:**

This single-blinded clinical trial was performed at School of dentistry, Shaheed Zulfiqar Ali Bhutto Medial University, Islamabad from 1st February, 2023 to 30th April, 2023. In this interventional study incorporated 120 participants with clinically visible signs of erosion, abrasion or recession. Visual Analog Scoring (VAS) Tool was used to investigate tooth sensitivity in these patients. Values of VAS for tooth sensitivity was calculated by exposing teeth of these patients to cold air blasting with triple syringe attached to dental unit at psi-30.0 pressure between 23±30ºC for duration of one second without using Azadirachta indica based Herbal mouthwash. Later on, these patients were provided with this Herbal mouthwash and its usage was recommended twice a day for one month. After One month, tooth sensitivity of patients was determined by VAS again. Data was analyzed by Paired T-test at 95% confidence and significance < 0.05.

**Results:**

VAS mean value for tooth sensitivity of patients before using *Azadirachta indica* based Herbal mouthwash was higher and found to be 55.43% ± 12.04 whereas its mean value after using Herbal mouthwash for one month reduced to 35.38% ± 11.62 which was statistically significant (*p* value=0.001). Reduction in tooth sensitivity of patients was almost 20.05% just after one month.

**Conclusion:**

*Azadirachta indica* based Herbal mouthwash was potent enough to reduce the tooth sensitivity in patients after one month of its usage.

## INTRODUCTION

Tooth sensitivity is the major global problem affecting most of the individuals irrespective of their age and gender but it enhances with aging especially during 3^rd^ and 4^th^ decades of life.^1^ The origin of this clinical condition is related to the dentinal tissue (dentinal tubules) exposure underlying the enamel portion of the tooth. Dentin hypersensitivity, designated as the chief concern of patients reporting to the dental clinic after caries and periodontal diseases. Enamel is the outermost protective shield of the tooth that guards the underlining pulp-dentin complex.^2^ Many etiological and predisposing factors contribute to the thinning and loss of the enamel surface of the tooth in turn adversely affecting the dentinal tubules underneath. The commonly involved factors responsible for this thinning of enamel include attrition, erosion, abrasion, and wear either due to hard brushing or acidic drinks. Once enamel gets thin and rough, it becomes capable of widening the dentinal tubules.^3^ Diameter of these dentinal tubules become more evident once they are opened after their expansion. Any irritant or stimulant released from the acidic beverages are easily absorbed into these widely opened dentinal tubules producing a sharp localized pain reaching the pulpal region of the affected tooth. The irritant or stimulant taking part in the tooth hypersensitivity could be chemical, thermal, tactile, osmotic, physical and mechanical.^2^,^3^ Moreover, these dentinal tubules provide the easily accessible passage to the bacteria and other microorganisms into the pulp thus, becoming the common source of infection in the pulp.^4^ Therefore, it is necessary to block these overly expanded dentinal tubules in order to bring them to their original smaller diameter. This could be the only possible solution to prevent the ingress of any irritant or stimulant into them hence, treating the tooth hypersensitivity and oral infections associated with it.

Currently, tooth sensitivity has been treated by distinct strategies such as: periodontally involved soft tissue grafting, fluoride ionotophoresis, homeopathic medicaments, Lasers, dentinal sealers, Ions and Salts.^5^ Although these strategies follow different treatment protocols but their main purpose to treat the tooth sensitivity could be achieved either through the nerve activity impairment or tubule occlusion.^6^ Toothpastes are the most simplest, reliable and cost effective means of treating the tooth sensitivity in patients suffering from this condition. The toothpastes mainly constituted of fluoride, strontium chloride, potassium oxalate, aluminum ferric oxalate, and stannous fluorides have the great tendency of occluding the dentinal-tubules on large scale.^7^ Potassium based salts and compounds containing toothpastes are most common for treating the tooth sensitivity.^8^ Various clinical studies have proven the efficacy of Potassium based toothpastes in relieving the tooth sensitivity and pain associated with this dental issue.^9^

Currently, the use of mouth wash has enormously enhanced to provide efficacious protection against dental plaque, carious lesions, and periodontal diseases. It is considered to be the most promising strategy for oral hygiene reinforcement. It contains antimicrobial agents, detergents, emulsifiers, and organic acids that strengthens its role against oral pathogens. Recently, many naturally procured herbal-based products have been tested and utilized in clinical dentistry to resolve the issues of dentin hypersensitivity. Herbal mouth rinses are a propitious source for managing dentin hypersensitivity but might lead to surface roughness^10^ till to date very little data pertaining the subject is available in the literature. The rationale of this trial was to treat the tooth sensitivity with the help of *Azadirachta indica-*based herbal mouthwash in patients. The hypothesis stated that the utilization of *Azadirachta indica-*based herbal mouthwash can effectively reduce the issues of dentin hypersensitivity in patents with tooth surface loss (wear).

## METHODS

This single-blinded clinical trial was performed at School of dentistry, Shaheed Zulfiqar Ali Bhutto Medial University, Islamabad.

### Ethical Approval:

The study was approved by the institutional ethical research committee (SOD/ERB/2023/22-04; dated: January 5, 2023). Duration of this study was three months from 1^st^ February, 2023 to 30^th^ April, 2023.

### Trial Registration:

This interventional study is recorded at *ClinicalTrials.gov* (ID: NCT06288776).

### Inclusion criteria:

-Participants aged 20 years and above in good general health with history of dentinal sensitivity associated with tooth surface loss (wear) within the past six months. No history of allergenicity. The involved teeth must be non-mobile, free of decay and restorations. Participants who have not used any mouthwash in last three month. A positive response to provoking air stimulus. The study teeth should have the clinically visible signs of tooth wear like: erosion, attrition or abrasion.

### Exclusion criteria:

-Participants who have used any medication that could alter pain perception within past two weeks, gross periodontal disease or periodontal surgery within past six months, multiple restored or decayed teeth and use of desensitizing toothpastes or tooth whitening treatment within two months of study were excluded from this trial.

### Sample size calculation:

Medical research requires precision and reliability; hence, sample size is important to study design. Clinical trials should be efficient and resource-optimized, according to Pocock (2013) trials examining treatment outcomes rarely require more than 100 to 200 patients. Following these suggestions, this study used Pourhoseingholi et al. (2013)’s formula to determine sample size, which is standard in medical research:


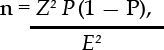


95% Confidence interval, Z=1.96 (corresponding to a 95% confidence level) P=0.5 (estimated proportion of the population with the attribute of interest) E=0.09 (desired precision level)


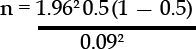


Replace the values in the calculation to get a sample size of 119 approximately. Thus, 120 samples were chosen for this study to ensure precision and statistical power.^11^,^12^

### Sampling technique:

Consecutive non-probability sampling technique was employed. All patients reporting to the Outpatient department of Dental hospital, School of Dentistry were assessed for the eligibility. Participants fulfilling the inclusion criteria were nominated for the intervention. This study enrolled 120 participants for the trial. They were duly briefed about the trial along with its probable merits and demerits. A written consent form was signed by each participant.

### Study Procedure:

Tooth sensitivity was evaluated by using Visual analogue Scale (VAS) system. VAS scoring system consists of a 100 mm scale where “0” at left side represented “Painless condition” while “100” at right side represented the “Worst pain condition”.^13^,^14^ The VAS values of these patients was checked as a confirmation of tooth sensitivity and the patients with VAS values 30-80 were selected in this study. The values of VAS were calculated by exposing the teeth of these patients to cold air blasting with the help of triple syringe attached to dental unit at psi-30.0 pressure between 23±30ºC for duration of one second before providing *Azadirachta indica* (NEEM) based Herbal mouthwash. Afterwards, these patients were provided with *Azadirachta indica* based Herbal mouthwash (Gumfit Mouthwash, Gennec Health, Sciences, PVT, LTD, Karachi, Pakistan) to use twice a day for 60 seconds for at least one month. In this clinical trial, name of the mouthwash was kept hidden from the participants involved. The natural ingredients present in this *Azadirachta indica* based Herbal mouthwash included *Azadirachta indica*, Aloe vera, Eucalyptus, Menthol, Methyl Salicylate, Sorbitol, Xylitol, Oregano extract, Glycerin, Clove oil, and Licorice extract. The patients were recommended to maintain their proper oral hygiene and avoid any type of dental treatment and other erosive food beverages to prevent any bias. These patients were recalled after one month of *Azadirachta indica* based Herbal mouthwash usage and their VAS scores were checked with the VAS scoring tool.^15^ The SPSS version-22 was utilized to calculate the mean and standard deviation values. The paired t-test at 95.0% confidence and *p* value < 0.05 was kept for the Significance.

## RESULTS

The intraclass correlation coefficient (ICC) was considered to measure the reliability, the single measures ICC was .757, 95% CI [.669, .824]. This indicates that approximately 75.7% of the variance in the scores can be attributed to differences between participants. This shows it is consistent and reliable measure of the construct in question.

**Fig.1:**
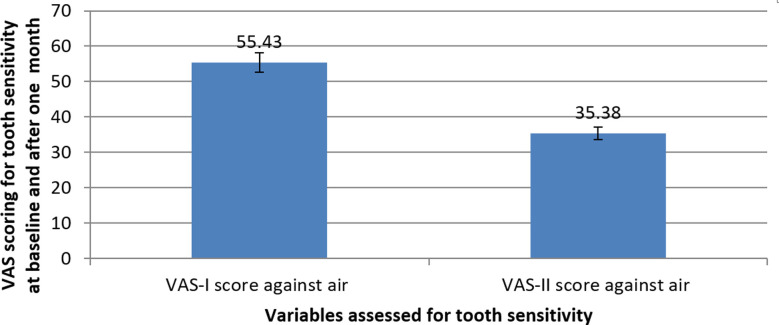
Mean difference between VAS values of patient’s tooth sensitivity before and after using the Azadirachta indica based Herbal mouthwash.

The results showed statistically significant reduction in the tooth sensitivity of the patients after using the *Azadirachta indica* based Herbal mouthwash for the period of one month. VAS tool calculated the mean value of tooth sensitivity of patients before using the *Azadirachta indica* based Herbal mouthwash which was found to be 55.43%±12.04 (1.09). This mean value of tooth sensitivity of patients after using the Herbal mouthwash for one month reduced to 35.38%±11.62 (1.06) which was statistically significant (*p* value ≤0.05) (Fig.1).

The difference in scores between baseline and one month after using the *Azadirachta indica* based Herbal mouthwash was evaluated using a paired samples t-test. A statistically significant difference between the two conditions was found in the results (t (119) = 26.629, p = .001). When compared to the mean score after using *Azadirachta indica* based Herbal mouthwash (M = 35.38%, SD = 11.62), the mean score before using Herbal mouthwash (M = 55.43%, SD = 12.04) was significantly higher. After using the *Azadirachta indica* based Herbal mouthwash for a month, the VAS scores for tooth sensitivity significantly decreased, with the 95% confidence interval for the difference in means spanning from 18.56 to 21.55 (Table-I).

**Table-I:** Paired t-test analysis showing 95% confidence interval before and after using *Azadirachta indica* based Herbal mouthwash with standard deviation (SD) and standard error (SE).

Group	Comparison of mean difference of VAS scoring before and after using *Azadirachta indica* based Herbal mouthwash for one month	S.D	S. E	95% Confidence Interval of the Difference		p-value

				Lower	Upper	
VAS scoring group	20.05%	8.25	0.75	18.56	21.55	0.001

## DISCUSSION

Natural biological plants and their extracts have been used widely in medicine and drug therapeutics because of their ease of availability, cost efficacy with least possible side-effects. Certain Medicinal plants play key role in combating various human diseases because of their unique features.^16^,^17^ The Neem plant (*Azadirachta indica*) is a medicinal plant found commonly in Pakistan and is very much effective in treating human diseases. The natural phytochemical components in the Neem plant are limonoid, sodium nimbinate, nimbidine, nimbin, quercetin, salanine, gedunine, and nimbolides which are famous for taking part in curing the human diseases.^18^ The antitumour, antibacterial, antiarthritics, hypoglycemic, antifungal, anti-inflammatory, antigastric ulcers, hypoglycemic, and antipyretic, properties of Neem are reported in various studies. The *Azadirachta indica (Neem plant)* was utilized in current study owing to its exceptional antimicrobial and medicinal properties.^19^-^22^

The current study utilized the Herbal mouthwash containing *Azadirachta indica* extract for treating the tooth sensitivity where it showed statistically significant declination in the tooth sensitivity of patients after the utilization of mouthwash for one month. The initial VAS value for tooth sensitivity before using the Herbal mouthwash was 55.43% displaying enhanced tooth sensitivity which became 35.38% after using the mouthwash for one month confirming the deduction in the tooth sensitivity of the patients (Fig.1). The reduction in VAS values for sensitivity to air in these patients was found to be 20.05% just after one which was quite effective and statistically significant (Tab) (p-value=0.001). The natural biomolecules found in the *Azadirachta indica* might have blocked the dentinal tubules and prevented the ingress of microorganisms and other toxic materials. Secondly, in case if any toxic material or bacteria entered the dentinal tubules the prominent medicinal properties of *Azadirachta indica* could have killed them thus, treating the tooth sensitivity, pain and pulpal infections. Henceforth, the incorporation of this herbal constituent into the mouthwash, owing to its medicinal properties and dentinal tubule occluding capacity, could possibly reduce dentin hypersensitivity in patients with tooth wear.^21^,^23^

The normal usage of herbal mouthwashes could be effective in preventing the gingival diseases, deposition of plaque^24^ and above all oral cancer.^25^ Several studies have shown encouraging results in favour of this trial. A study supported the medicinal impact of herbal mouth washes by reporting the active role of *Azadirachta indica* based mouthwash in treating the gingival and periodontal diseases in the clinical dentistry.^23^ Another researcher has successfully utilized the mouthwash formulation containing *Acacia Arabica* that cured gingivitis in the patients effectively^26^, and in another research *Acacia Arabica* plant resolved the tooth sensitivity to some extent.^27^ Other studies confirmed that bark of cinnamon and clove were helpful in reducing the tooth sensitivity. Herbal ingredients present in plants display strong action in preventing the tooth destruction including decay, sensitivity, ulcers, and discoloration.^28^,^29^ Thus, current study confirmed that the inclusion of *Azadirachta indica* in the Herbal mouthwash was potent enough to treat the dentin hypersensitivity being a valuable medicinal plant available.

Herbal-based dental products are an advancement in clinical dentistry. By embodying the medicinal properties of herbal products in commercially available over the counter products, it can prove to be boons to modern clinical practice. The inclusion of herbal constituents into the mouthwash has produced satisfactory results in terms of tooth sensitivity thus supporting the hypothesis that utilization of *Azadirachta indica* herbal-based mouth wash can effectively reduce the issues of dentin hypersensitivity in patients due to tooth surface loss (wear). Thus, it opens gateways for further research work in this domain to provide evidence-based data in support of medicinal plants in clinical practice.

### Limitations:

Nonetheless, the study has certain limitations as well. The one month evaluation period, although feasible for both the investigator and the patients, could be enhanced to authenticate the validity of this trial. Furthermore, it could possibly shed light on the long term benefits of using herbal mouth washes.

## CONCLUSION

*Azadirachta indica* in the Herbal mouthwash played a significant role in reducing the tooth sensitivity of about 20.05% in the patients as compared to the values before using mouthwash. This showed that being medicinal plant *Azadirachta indica*, by occluding patent dentinal tubules, was strong enough in relieving the tooth sensitivity to greater extent and could be recommended for regular usage in order to maintain the oral hygiene by preventing the oral diseases.

### Authors’ Contribution:

**AM and EM:** Conceptualization and methodology. **AM and KH:** Data Analysis & interpretation, review & editing and critical review. **AM, EM and NA:** Data collection, data processing and original draft. **AM** is responsible for the accuracy of study.
